# Cocktail strategy based on a dual function nanoparticle and immune activator for effective tumor suppressive

**DOI:** 10.1186/s12951-022-01241-y

**Published:** 2022-02-17

**Authors:** Qian Li, Qiubing Chen, Xue Yang, Yuelan Zhang, Linyue Lv, Zhuyou Zhang, Shaowei Zeng, Jiaxi Lv, Sijin Liu, Bishi Fu

**Affiliations:** 1 Department of Paediatrics, State Key Laboratory of Virology, Frontier Science Center for Immunology and Metabolism, Medical Research Institute, Zhongnan Hospital of Wuhan University, Wuhan, China; 2 Department of Pulmonary and Critical Care Medicine, Zhongnan Hospital of Wuhan University, Wuhan, China; 3 Wuhan Research Center for Infectious Diseases and Cancer, Chinese Academy of Medical Sciences, Wuhan, China; 4 Department of Clinical Medicine, Fourth Clinical Medical College, Capital Medical University, Beijing, People’s Republic of China; 5 State Key Laboratory of Environmental Chemistry and Ecotoxicology, Research Center for Eco-Environmental Sciences, Chinese Academy of Sciences, Beijing, China

**Keywords:** cGAS/STING, Metabolism inhibitors, Hepa1–6 cells, STING agonists

## Abstract

**Background:**

Immune checkpoint inhibitor-mediated immunotherapy cannot be carried out on a large scale clinically due to its low universality. In recent years, cyclic guanosine monophosphate synthase/interferon gene stimulating factor (cGAS/STING)-mediated innate immune signaling pathway-mediated immunotherapy has attracted more and more attention. In addition, metabolic inhibitors also show good effects on tumor treatment, but their application is often limited because of their large first pass effect or difficult administration.

**Methods:**

The particle size and potential parameters were measured by DLS. In order to determine the optimal ratio of the two drugs, we calculated the CI value of different nanoparticles through MTT experiment, and simulated their synergistic effect through Gaussian software. Then the morphology and crystal form of the best proportion of drugs were studied by TEM and XRD. The anti-tumor mechanism of composite nanoparticles was confirmed by the determination of metabolic related indexes, Q-PCR and WB. The antitumor effect and immune activation effect were comprehensively evaluated by in vivo and in vitro experiments.

**Results:**

Here, we found and synthesized BCP nanoparticles ((BPA + CPI) @ PLGA NPs) which can effectively reduce the metabolism of tumor cells and inhibit cell proliferation. At the same time, the release of mitochondrial DNA (mtDNA) caused by mitochondrial metabolism disorder further activated the cGAS/STING signal pathway in Hepa1–6 cells. We found that the drug-treated Hepa1–6 cells had obvious TBK1 phosphorylation and STING dimerization. Combined with STING agonist, it could effectively promote the activation of CD8 T cells and enhanced the therapeutic effect on liver cancer.

**Conclusion:**

Our results showed that PLGA nanocarrier can successfully improve the dosage forms of two metabolic inhibitors and show the effect of synergistic therapy. BCP nanoparticles can also activate the innate immunity of tumor cells and significantly enhance tumor inhibition after combined with STING agonists. This study has high reference and transformation value for the combined treatment of immunosuppression and metabolic inhibition.

**Graphical Abstract:**

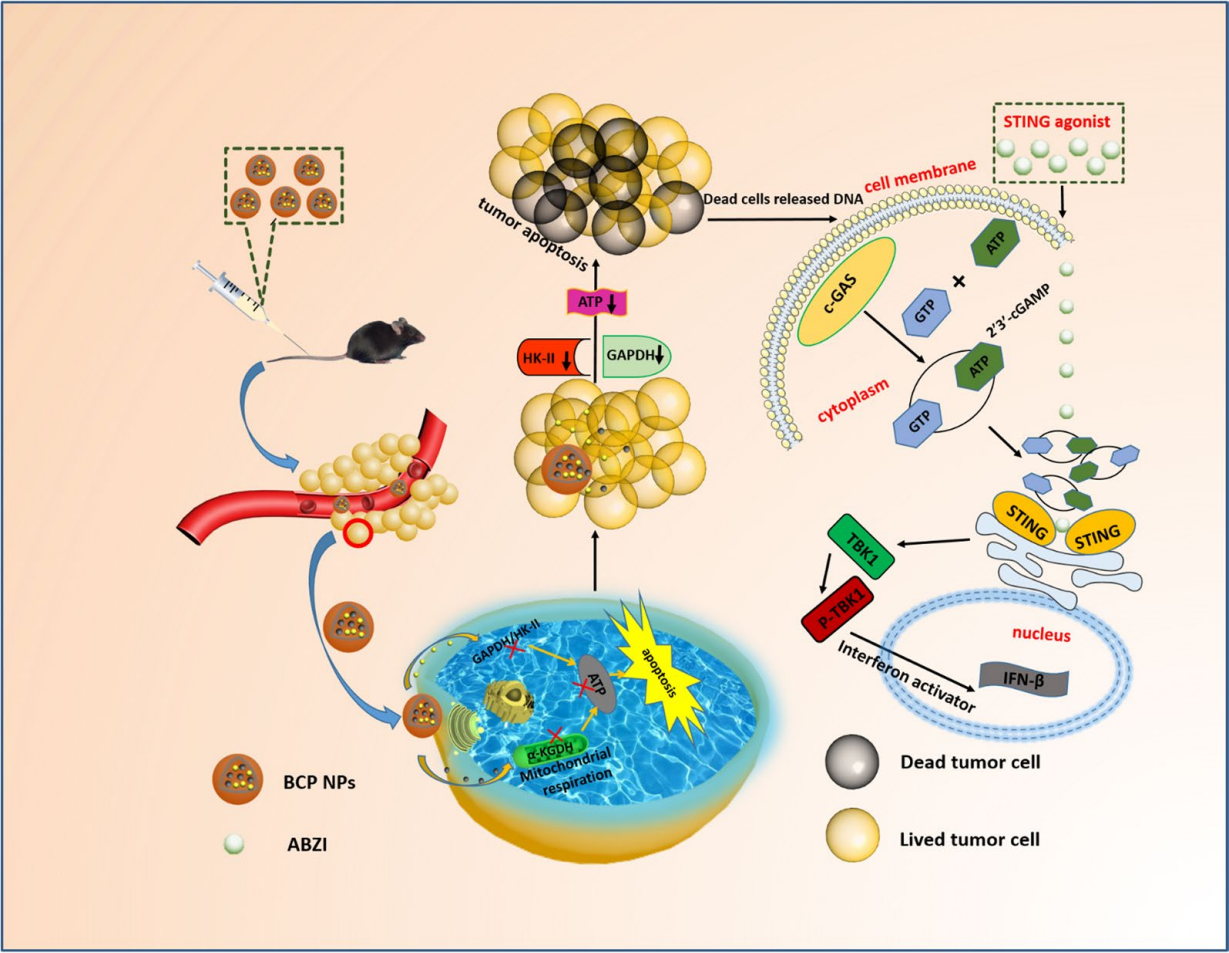

**Supplementary Information:**

The online version contains supplementary material available at 10.1186/s12951-022-01241-y.

## Introduction

Tumor growth needs massive ATP as energy supply. And the ability of tumor cells to absorb grapes is dozens of times higher than that of normal tissue cells, which called the "Warburg effect" [1–3]. Even under aerobic conditions, glucose produces lactic acid in tumor cells through glycolysis. It is a new treatment strategy for anti-tumor therapy by targeting tumor energy metabolism currently [4–6]. Among them, 3-bromopyruvate (3-BPA) and CPI-613 are widely studied as metabolic blockers [7–10]. 3-BPA has high affinity with GADPH and HK-II [11, 12]. It can completely interdict the activities of GADPH and HK-II under very low concentration [13, 14]. However, 3-BPA is instable in the liquid state and easy to decompose [15, 16]. CPI-613 is an inhibitor of mitochondrial tricarboxylic acid cycle metabolism in tumor cells [17, 18]. It suppresses the α-ketoglutarate dehydrogenase complex (KGDH) to restrain the glutamine replenishment pathway [19, 20]. Whereas CPI-613 is lipid-soluble which easily soluble in organic solvents, and difficult to dissolve in water [21, 22].

In recent years, with the rapid development of nanotechnology and wide application, nanomaterials have been widely used in biomedicine. Nanoparticles can protect drugs, reduce drug systemic toxicity, improve drug uptake, slow-release, and targeted drug delivery [23–25]. Hence, the above problem of drug solubility and stability in application could be well resolved by using nanomaterial.

Cancer immunotherapy is another new approach of anti-tumor therapy that apply the host immune system to fight against cancer [26]. Stimulator of interferon genes (STING), also known as transmembrane protein 173 (TMEM173) and MPYS/MITA/ERIS, which can induce the production of a variety of pro-inflammatory cytokines and chemokines, is shown great potential in enhancing anti-tumor immunity [27]. Many STING agonists have been developed or tested in preclinical and clinical trials for immunotherapy of diseases such as cancer and infectious diseases [28–30]. And yet, In terms of the current clinical attempts, it mainly focus on modified cyclic dinucleotide (CDN) to simulate the STING’s endogenous ligand (cGAMP) to treat solid tumors by intratumoral injection [29]. Using the symmetry of STING, GSK designed a connection strategy to cooperate with two symmetrically related amide benzimidazole (ABZI) based compounds to produce a high affinity ligand that interacts with STING in a cGAMP competitive manner. What’s more, ABZI was the first nonnucleotide intravenous STING agonist recently. ABZI not only overcomes the disadvantages of intratumoral injection but also has a strong anti-tumor effect [30].

In this study, 3-BPA and CPI-613 are applied conjunctively to block the energy metabolism of Hepatocellular carcinoma (HCC) via a nano-drug delivery system for the first time. Meanwhile, we combine BCP NPs with anti-tumor immunotherapy innovatively. Our study sweeps the obstacle of poor solubility and easy decomposition in metabolic drugs combination. Moreover, paves a novel way to effectively restrict the growth and metastasis of HCC. We also provide a reliable basis for future research on the combined administration of metabolic and immune therapy.

## Results

### BCP NPs synthesis and characterizations

**Scheme 1:**
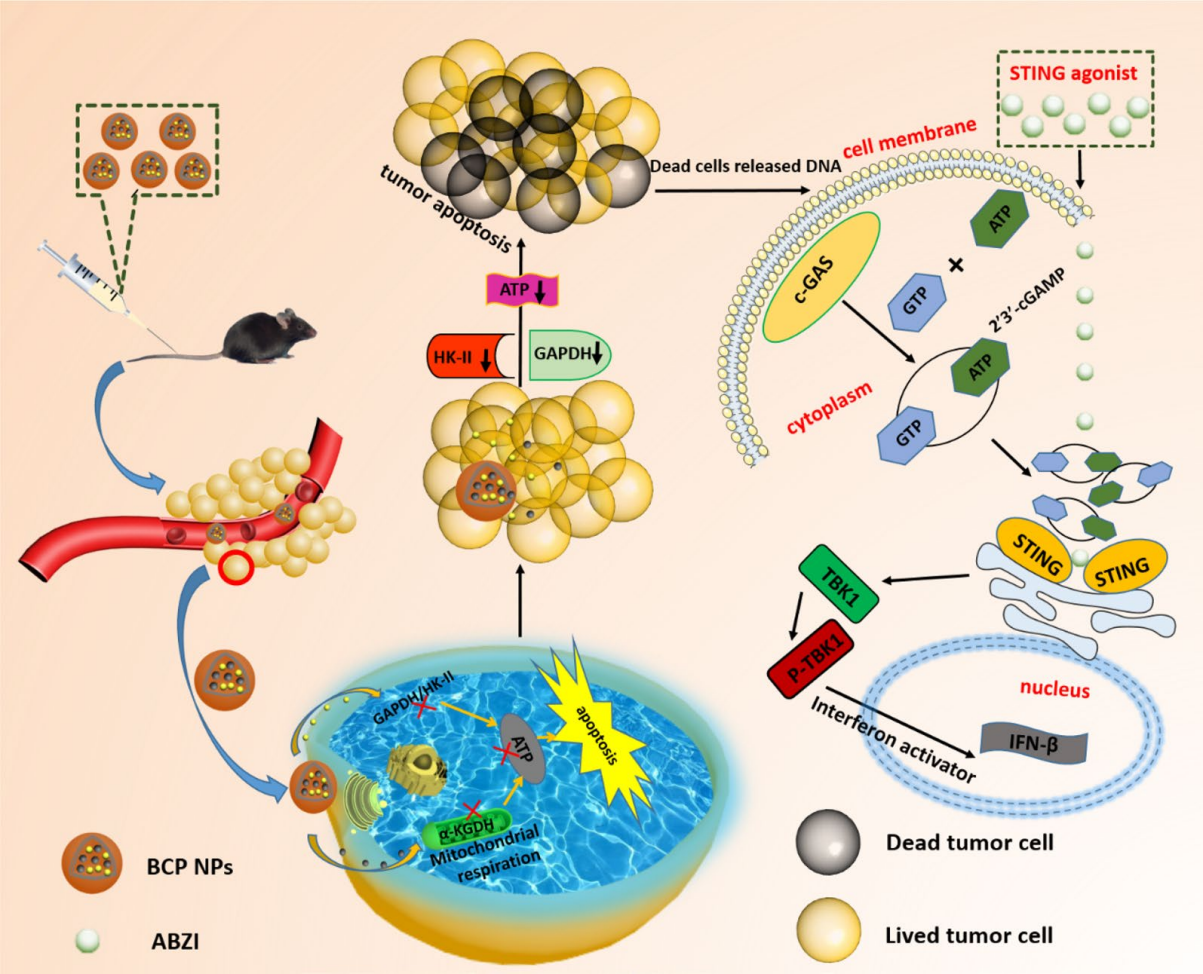
Illustration of the the anti-tumor mechanism of BCP NPs

The BCP NPs synthesis procedure and its anti-tumor mechanism is illustrated in Scheme 1. The PLGA NPs were prepared by an emulsion solvent evaporation approach. Meanwhile, CPI-613 and 3-BPA were regarded as oil phase and water phase respectively, loaded in the PLGA NPs. A dynamic light scattering (DLS) was applied to measure the size and zeta potential of BCP NPs (Additional file 1: Table S1). The particle size and potential of different molar ratio BCP NPs were not much different. High performance liquid chromatograph (HPLC) and pyruvate assay kit were used to determine the drug load and encapsulation rate of the different BCP NPs (Additional file 1: Table S2).

**Fig. 1:**
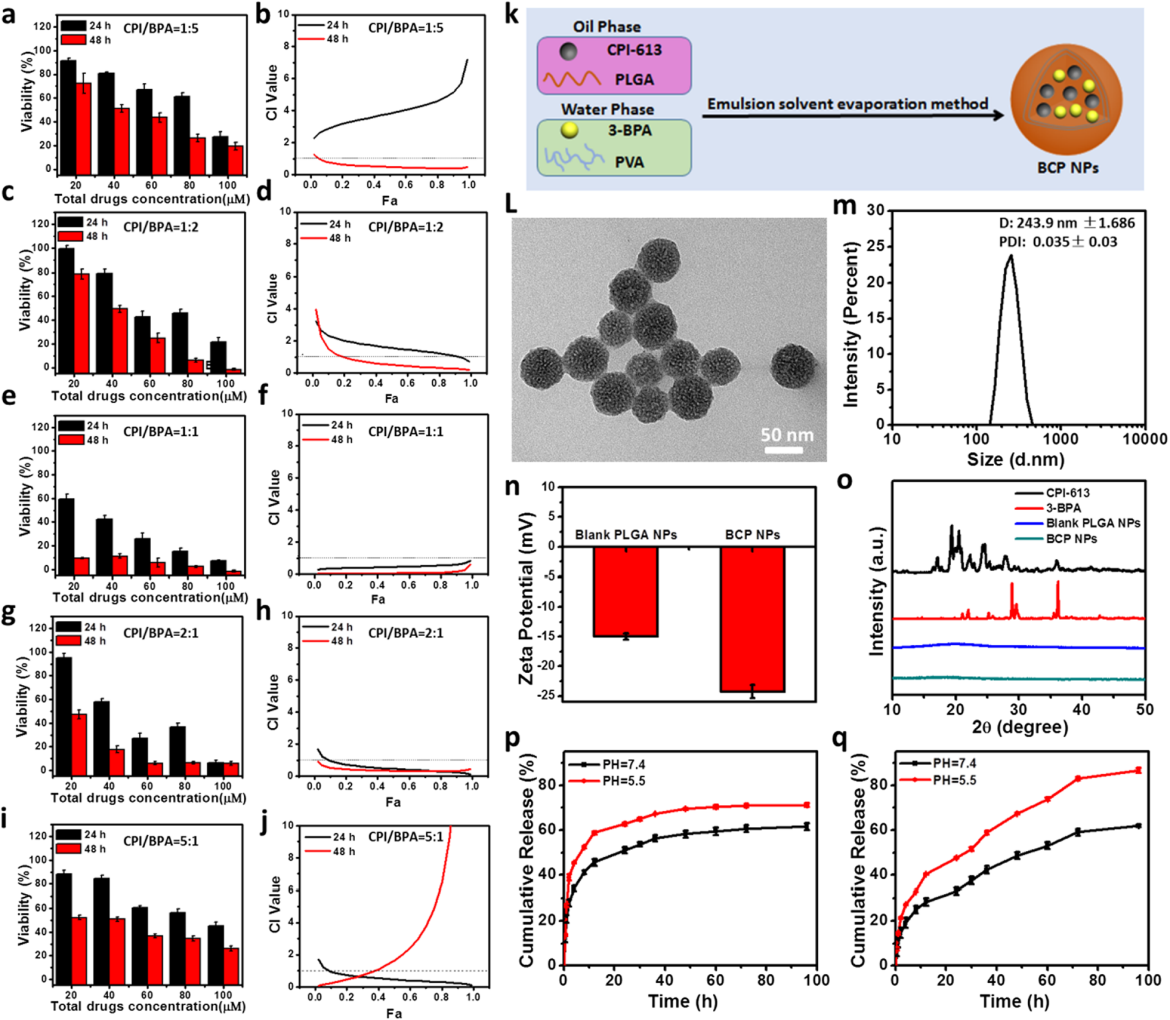
In vitro cytotoxicity of CPI/BPA (1:5) (**a**), CPI/BPA-NP (1:2) (**c**), CPI/BPA (1:1) (**e**), CPI/BPA (2:1) (**g**), CPI/BPA (5:1) (**i**) against Hepa1–6 cells, determined by MTT assays; CI versus Fa plots for CPI/BPA (1:5) (**b**), CPI/BPA-NP (1:2) (**d**), CPI/BPA (1:1) (**f**), CPI/BPA (2:1) (**h**) and CPI/BPA (5:1) (**j**); Synthesis process of BCP nanoparticles (**k**); TEM images of BCP NPs (**l**); size distribution of BCP NPs measured by DLS (**m**); Zeta potentials of Blank PLGA NPs and BCP NPs (**n**); XRD of CPI-613,3-BPA, Blank PLGA NPs and CBP NPs (**o**); Cumulative Release of Cy-7 (**p**) and Coumarin (**q**) in PLGA NPs

**Fig. 2:**
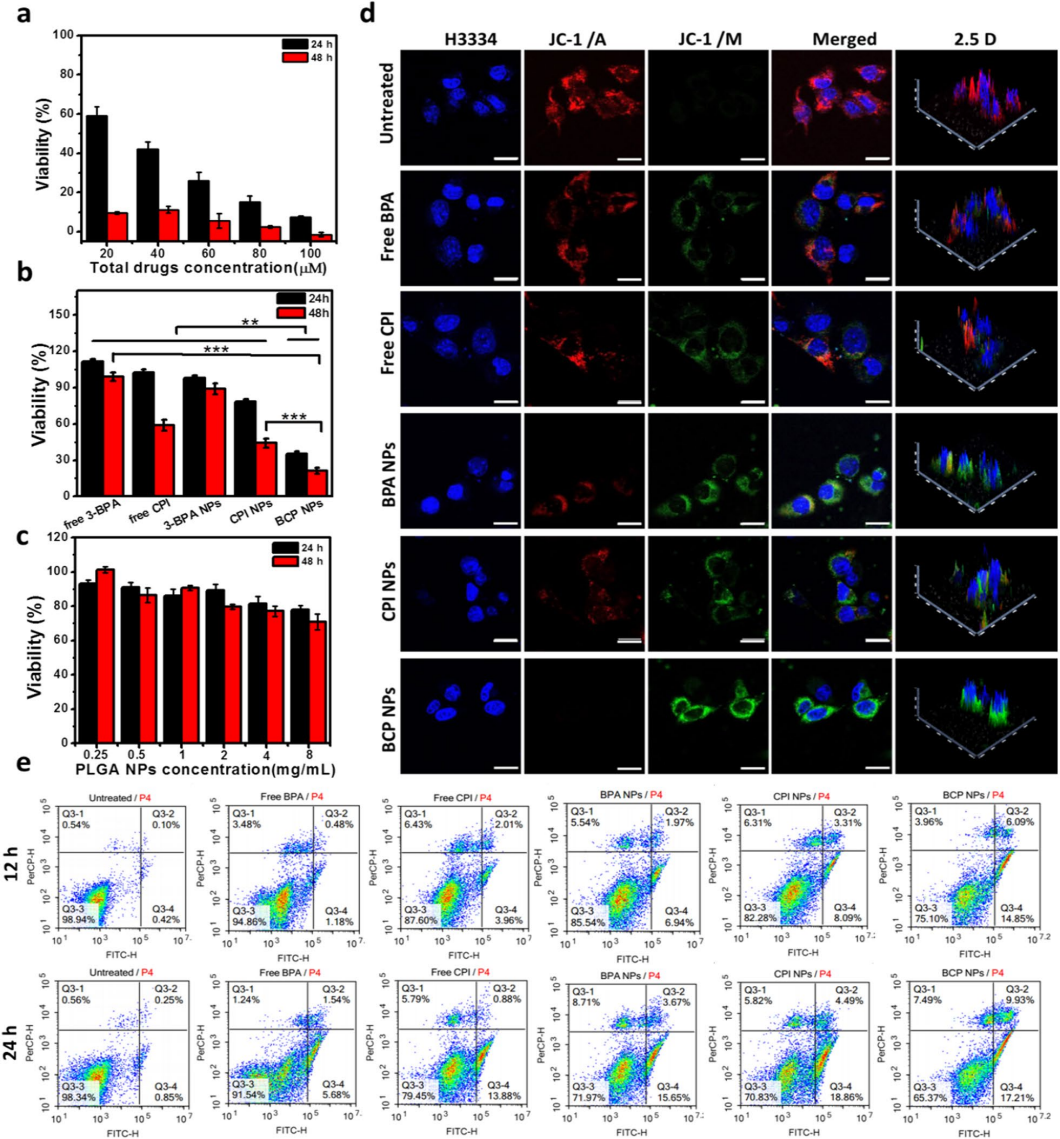
Viability of Hepa1–6 cells treated with BCP NPs under gradient concentrations (**a**) and different drugs for 24 h and 48 h (**b**); viability of Hepa1–6 cells incubated with blank PLGA NPs at different concentrations for 24 h and 48 h (**c**); Fluorescence images of Hepa1–6 cells tumor cells after various treatments analyzed by JC-1 staining (scale bar: 20 µm, JC-1/A and JC-1/M signified the aggregate and monomer form of JC-1 (**d**); Flow cytometry analysis of cell apoptosis by staining with Alexa Fluor 488 annexin V-FITC and PI after various treatments (**e**)

As reported, the synergistic effect of two or more drugs largely depended on a series of factors, such as combination ratios, drug concentrations, and treatment time etc. [31]. Therefore, the effects of different molar ratios, total drug concentration and different treatment times were investigated (Fig. 2). The BCP NPs has obvious killing activity to Hepa1–6 cells in a dose-dependent manner. In addition, the cytotoxicity was the strongest while the drug molar ratio as 1:1. CI value and IC_50_ of different molar ratios were calculated to assess the synergistic effect of the two drugs by using CalcuSyn software (Biosoft, UK) (Fig. 1a–j, Additional file 1: Table S3). The CI value of BCP NPs is less than others and IC_50_ value is the lowest while the molar ratio of the two drug is 1:1, and it has significant synergistic effect (CI value > 1, Antagonism effect; CI value = 1, Combined effect; The CI value < 1, Synergy effect) at this ratio. Therefore, the particles with a molar ratio of 1:1 were chosen for the following experiments. A good anticancer drug must have good tumor specificity [32]. Therefore, we selected normal hepatocyte AML12 for MTT experiment (Additional file 1: Fig. S1). The results showed that BCP nanoparticles had no obvious toxicity to normal hepatocytes. This may be due to more vigorous metabolism in tumor cells [33, 34]. This showed that the BCP nanoparticles can specifically kill tumor cells without obvious toxic and side effects. At the same time, we also studied the inhibition of BCP nanoparticles on other tumor cells (Additional file 1: Fig. S2). We found that BCP nanoparticles had obvious toxicity to 4T1 cells and MCF-7 cells. The results were consistent with Hepa1–6 cells in a concentration dependent and time-dependent manner. This also showed that the BCP nanoparticles we synthesized have certain universality in terms of tumor lethality.

The surface topography of BCP NPs was explored by transmission electron microscopy (TEM). The TEM of BCP NPs showed a uniform spherical shape (Fig. 1l). The size and PDI value of BCP NPs were measured as 243.9 ± 1.686 nm and 0.035 ± 0.03 nm, respectively. It was slightly larger than obtained from TEM imaging due to the colloidal hydration (Fig. 1m). This may be due to the DLS test was carried out in aqueous solution, which would form nano clusters and may had slight agglomeration. DLS reflected its average particle size in aqueous solution. TEM was tested in a completely dry environment, which reflects the real size of the nanoparticles, which was also described in the previous studied [35, 36]. The zeta potential of Blank PLGA NPs and BCP NPs were − 14.97 ± 0.54 mV and − 24.27 ± 1.11 mV, respectively (Fig. 1n). XRD pattern further shown that free CPI-613 and 3-BPA both have distinct crystal forms. Meanwhile, the Blank PLGA NPs and BCP NPs have no crystalline form. It may be that the interaction between CPI-613/3-BPA and PLGA polymer molecules forms an amorphous complex. Therefore, this result shows that the drug is encapsulated in the polymer in molecular form, which is consistent with the previously published report [37] (Fig. 1o). It implied that the CPI-613 and 3-BPA were loaded in the PLGA NPs, and the crystalline form was covered. Furthermore, long-term stability of BCP NPs in different media were tested. DI water, PBS, and DMEM with 10% FBS were evaluated over 7 days based on the changes of particles size by DLS (Additional file 1: Fig. S3). The hydrodynamic size of BCP NPs had no dramatic change in the different conditions, indicating the BCP NPs has a good colloidal stability.

The mode dyes hydrophilic Cy7 and Coumarin 6 were selected to qualify the drug release properties of nanoparticles (Fig. 1p, q). The results showed the release rate of Cy7 and Coumarin 6 were more fast in pH5.5 buffer solution than in pH7.4 buffer solution, which implied the PLGA had a faster degradable rate in low pH environment. This hypothesis has been reported in previous articles [38–40]. The release rate of Cy7 was faster than Coumarin 6 in nanoparticles due to th Cy7 was hydrophilic but the Coumarin 6 was hydrophobic. It also shown that both Cy7 and Coumarin 6 showed a trend of rapid released at the initial stage, and then relatively slowed release [31], which consistent with previous reports [41]. Coumarin 6 was also selected to determine the intracellular uptake ability of nanoparticles. The data also shown that intracellular uptake was a gradual process. And the same result from of the Flow analysis (Additional file 1: Fig. S4).

### Anti-tumor activity of BCP NPs in vitro

The BCP NPs anti-tumor efficacy was further evaluated in vitro. As shown in Fig. 2a, the BCP NPs performed a significant anti-tumor capability in Hepa1–6 cells. It boosts the anti-tumor capability as the increase of particle concentration. In particularly, when the total drugs concentration was 60 µM, the cellular viability of 24 and 48 h were 26.14% and 5.57% respectively (Fig. 2a). We also tested the cell toxicity of individual drug by MTT assay (the same amount of total drug concentration: 50 µM). Apparently, the BCP NP significantly increased the cytotoxicity to Hepa1–6 cells compared with CPI NPs, 3-BPA NPs and free drugs (Fig. 2b). Meanwhile, the blank PLGA NPs did not show any cytotoxicity for Hepa1–6 cells (Fig. 2c).

Moreover, cellular fluorescent staining experiments further indicated that the BCP NPs treated group was more effective in killing Hepa1–6 cells than other groups (Additional file 1: Fig. S4).

**Fig. 3:**
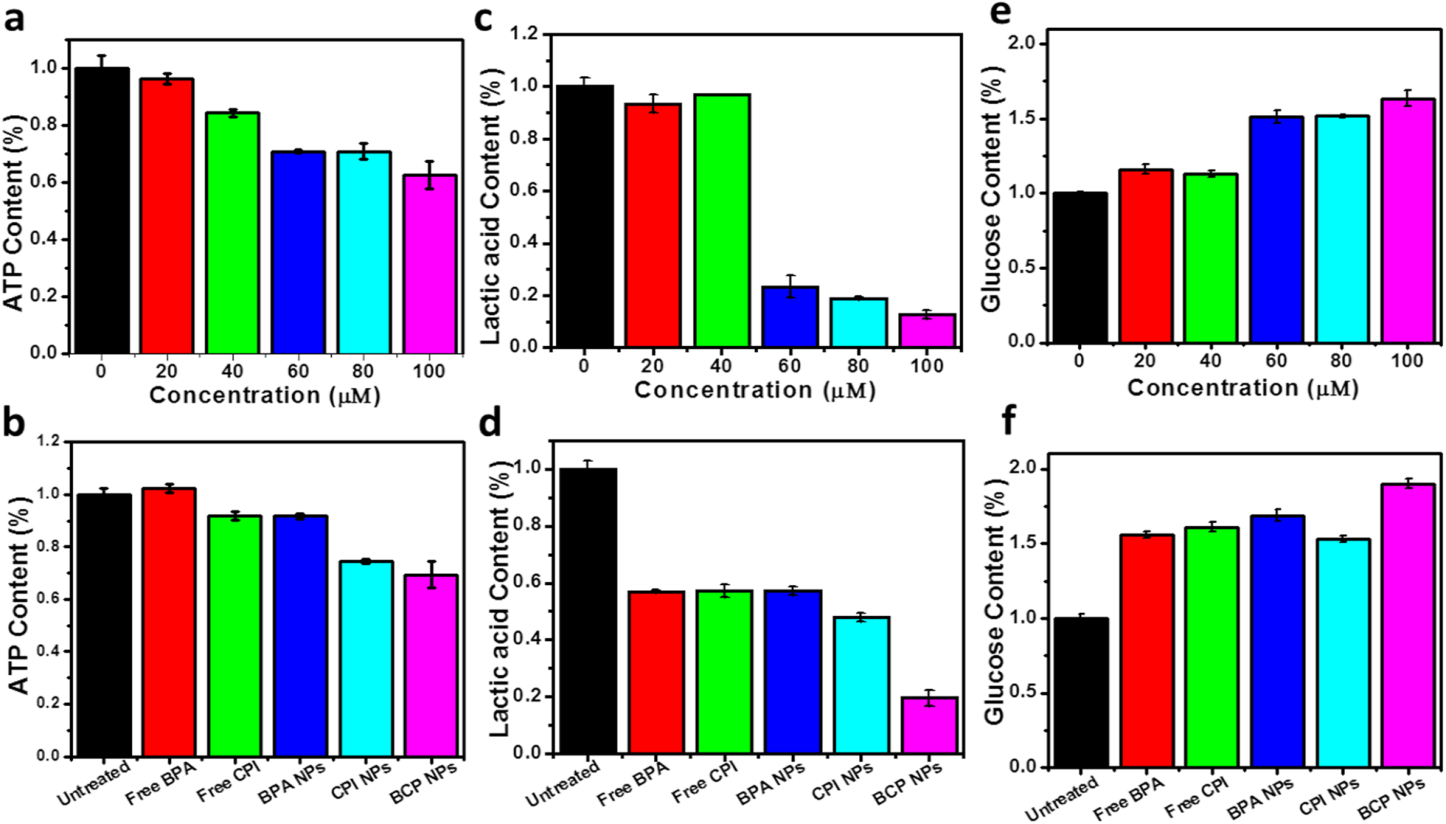
Intracellular changes of ATP (**a**, **b**); lactic acid (**c**, **d**) and gluconolactic acid (**e**, **f**)

The dysfunction of mitochondria and cell viability are closely related. And the variation of mitochondrial membrane potential (MMP) can be visualized by the JC-1 probe [42–44]. As can be seen in Fig. 3d, the untreated group showed strong red fluorescence, indicating normal mitochondrial function. When cells were exposed to free 3-BPA and free CPI-613, a slight green fluorescence was monitored. Meanwhile, the 3-BPA NPs and CPI-613 NPs groups showed a brighter green fluorescence, indicating the MMP decreased with administration. However, the cells treated with BCP NPs showed the strongest green fluorescence (Fig. 2d). The above results suggested that the BCP NPs group possess the strongest cell killing ability, which was consistent with results of cytotoxicity assays, *Annexin V-Alexa Fluor* *488* FITC and PI staining and live/dead staining (Fig. 2e, Additional file 1: Fig. S5).

### BCP NPs restricts cellular metabolism gene expression

The intracellular ATP content, lactic acid secretion rate and glucose absorption rate were detected after treatment with different drugs concentrations and diverse drugs in Hepa1–6 according to the instructions. The results showed that the BCP NPs could strongly inhibit the production of ATP, lactic acid secretion and glucose absorption of Hepa1–6 cells. What’s more, the inhibitory ability was enhanced while increasing of drug concentration (Fig. 3a–c). Compared with untreated group, all tested groups had certain inhibitory ability. And the BCP NPs group had the highest inhibition ratio to the production of ATP, lactic acid secretion and glucose absorption of Hepa1–6 cells. All those indicated that BCP NPs have a more effective potential to inhibit cell metabolism (Fig. 3d–f).

**Fig. 4:**
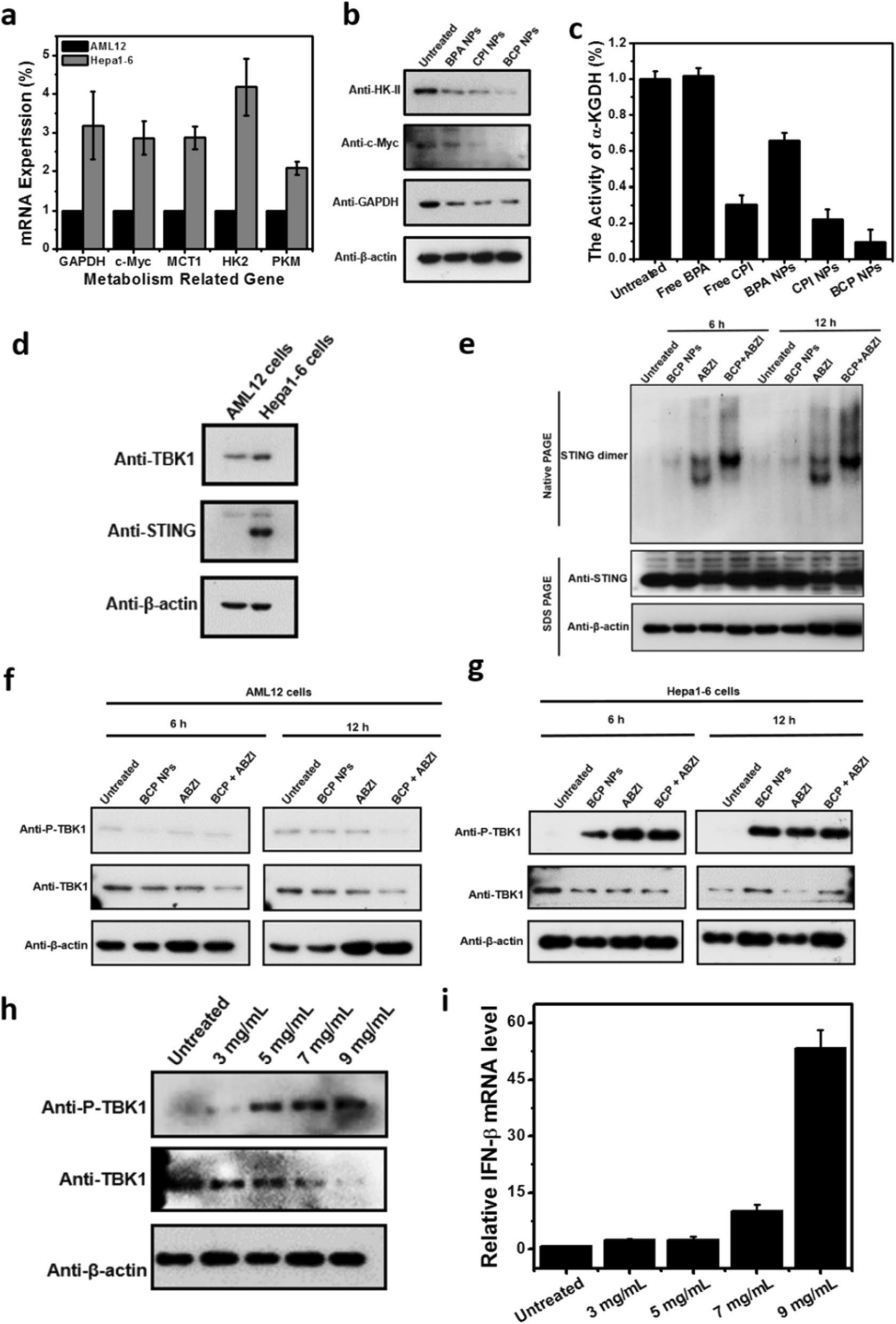
The mRNA expression of metabolism-related genes of AML12 cells and Hepa1–6 cells (**a**); Western blot analysis of HK-II, c-Myc and GAPDH. β-actin was used as an internal reference (**b**); The activity of α-KGDH (**c**); The Western blot analysis of TBK1 and STING in AML12 cells and Hepa1–6 cells (**d**); Effects of BCP NPs, ABZI and BCP NPs + ABZI treated Hepa1–6 cells triggered dimerization of STING (**e**); Effects of BCP NPs, ABZI and BCP NPs + ABZI treated Hepa1–6 cells triggered phosphorylation of TBK1 in AML12 cells (**f**) and Hepa1–6 cells (**g**); Different concentration BCP NPs treated Hepa1–6 cells triggered phosphorylation of TBK1 in Hepa1-6 cells (**h**); The relatived production of IFN-β after the treatment of different concentration BCP NPs treated Hepa1–6 cells (**i**)

In order to verify either BCP NPs have influence to ‘Warburg effect’, metabolism related genes expression levels were compared in normal hepatocytes AML12 and Hepatoma cells, Hepa1–6. The expression levels of GAPDH, c-Myc, MCT1, HK-II, PKM, LDHA in Hepa1-6 cells were significantly higher than those in AML12 cells, which was consistent with the Warburg theory (Fig. 4a). After treated with diverse drugs to Hepa1–6, the expression of related metabolic genes decreased compared with untreated group. The decrease trend was most obvious in BCP NPs group compared with other control groups (Fig. 4b and Additional file 1: Fig. S6). Meanwhile, the activity of α-KGDH implied that the compared with BPA NPs and CPI NPs groups, the protein expression of c-Myc, GAPDH and HK-II in BCP NPs treated group decreased most significantly (Fig. 4c). The above results demonstrate that BCP NPs had the strongest restriction on the metabolism related genes expression of Hepa1–6 cells.

### BCP NPs and innate immunity

In order to study the mechanism of tumor cell death caused by BCP NPs, we further validated either BCP NPs stimulate the innate immunity. Firstly, we were surprised to find that STING was highly expressed in Hepa1–6 cells, while normal hepatocytes AML12 cells contained little or no STING (Fig. 4d). Subsequently, ABZI, a STING agonist [30], was selected as the positive control to detect STING dimerization in Hepa1–6 cells after treatment with BCP NPs. The results showed that STING dimerized significantly after treatment with BCP NPs for 6 h or 12 h. respectively (Fig. 4e). This result suggested that BCP NPs could activated STING as agonists. As previously reported, After cGAs enzyme senses DNA, it will activate cGAMP, then STING was activated, and finally produce type I interferon and proinflammatory cytokines [45–47]. And the cGAs enzyme can be activated not only by extracellular DNA and DNA viruses, but also by cytoplasmic DNA (such as mitochondrial DNA). In addition to the external stimulation, extracellular DNA may also come from the DNA released by surrounding cells [48]. Therefore, we speculate that BCP treatment of Hepa1–6 cells may lead to the death of some cells, and the DNA released by dead cells activates sting of surrounding cells. Subsequently, we detected the phosphorylation of TBK1 in AML12 cells and Hepa1–6 cells after treatment with BCP NPs. The results showed that TBK1 was significantly phosphorylated in Hepa1–6 cells but not in AML12 cells (Fig. 4f–h). In addition, BCP NPs treated Hepa1–6 cells produced more IFN-β (Fig. 4i). These results implied that the BCP NPs could activate STING signal pathway and further activate anti-tumor innate immunity.

### BCP NPs’ safety evaluation in vitro

In order to verify the blood compatibility of BCP NPs in vitro. Red blood cells separated from healthy mice incubated with BCP NPs for a certain time. The hemolysis rate of red blood cells was always less than 5% while treated with different concentration of BCP NPs, even up to 100 μM. These results suggested that BCP NPs have good blood compatibility (Additional file 1: Fig. S7).

**Fig. 5:**
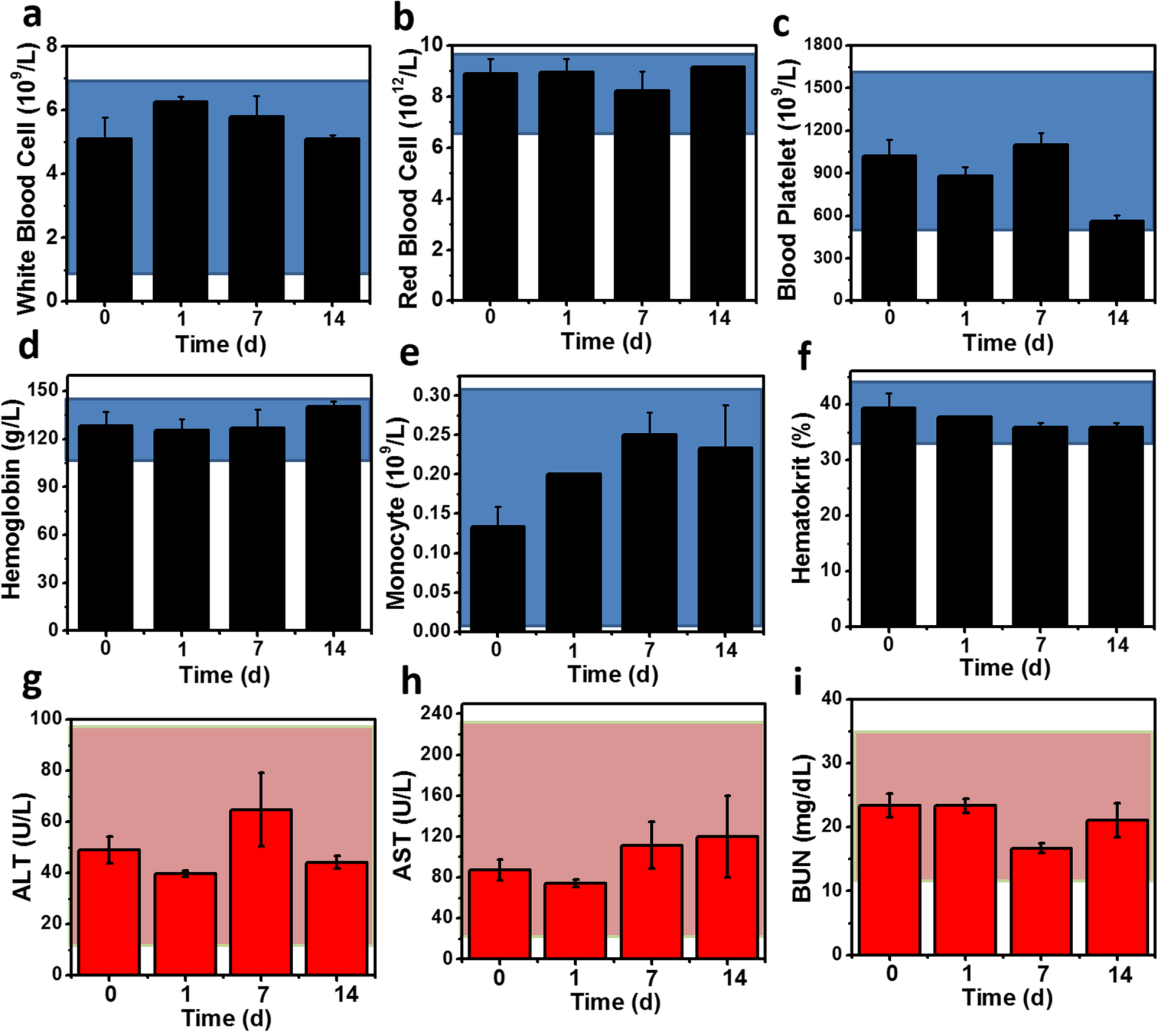
Primary indicators of blood routine test (**a**–**f**) and liver and kidney function (**g**–**i**) after C57BL/6 mice treated with or without BCP NPs. The blue pink hatched areas represent the reference ranges of hematology index of healthy mice

The toxic and side effects of BCP NPs was evaluated further in vivo. The blood constants and biochemical indexes was measured after administration. Briefly, mice were injected BCP NPs intravenously, the blood samples were collected on the 1st, 7th and 14th days respectively. The blood index, liver and kidney function of mice were evaluated by whole blood cell count and blood biochemical analysis after administration. None of abnormal functional marker can be found in result and all index were normal (Fig. 5). In parallel, the mice tissues and different organs were harvested at 14 days for H&E staining. Compared with control, the heart, liver, spleen, lung, kidney and other organs of the mice in BCP injection group had no obvious pathological variation, suggested that BCP NPs had no obvious toxic and side effects to major organs in vivo (Fig. 5).

### In vivo anti-tumor effect of BCP NPs

**Fig. 6:**
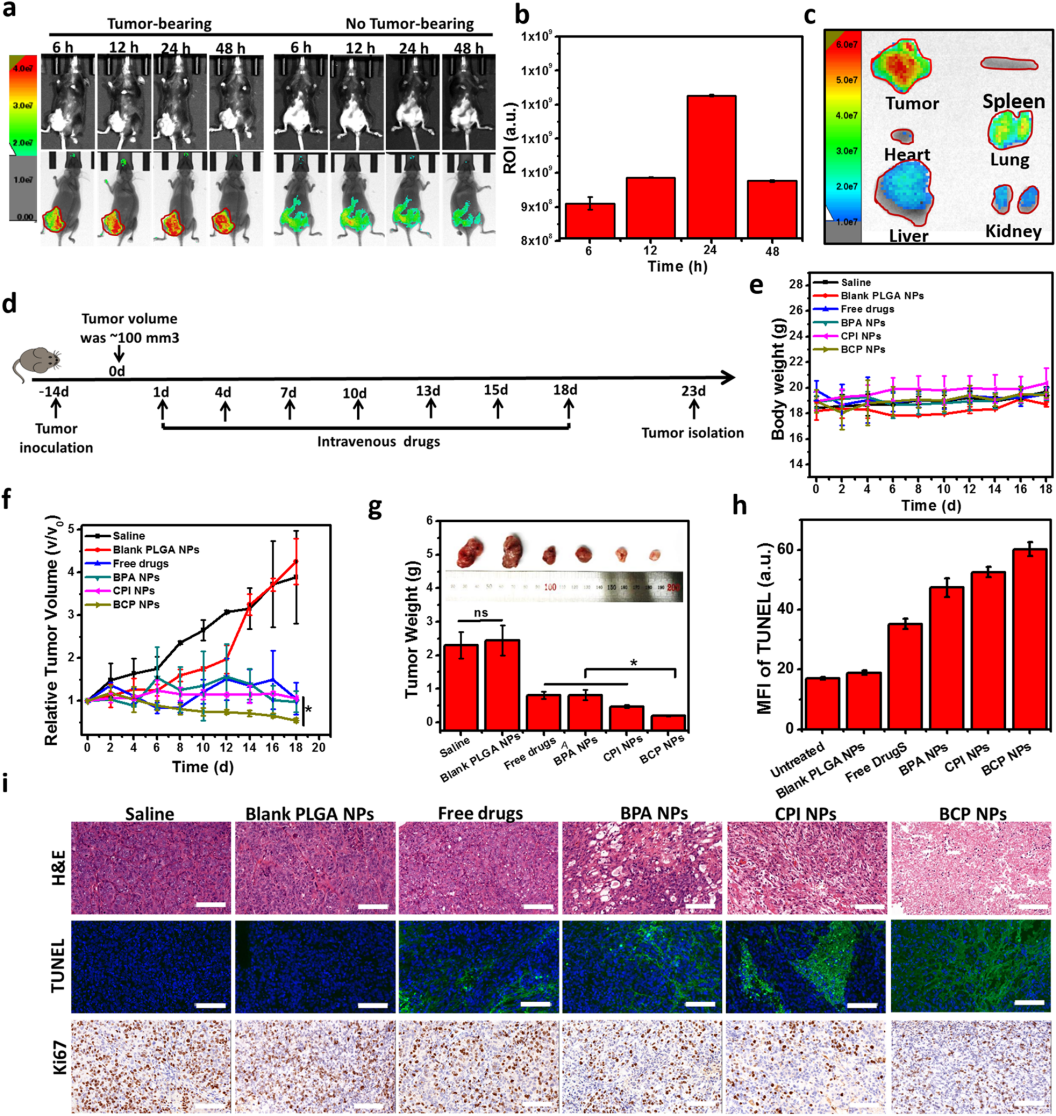
Fluorescence images of C57BL/6 mice bearing Hepa1–6 tumors at different time points after the injection of BCP NPs, and the fluorescence images of tumors and different organs excised at 48 h post-injection (**a**–**c**); Schematic of treatment on a Hepa1–6 tumor model (**d**); Body weight over 18 days after the injection of different treatment (**e**); Tumor volume variation after different treatments (**f**); The weight of different resected tumors on day 18 (**g**); Mean fluorescence intensity of TUNEL section staining (**h**); Tumor tissue section analysis of tumor sections by H&E staining,TUNEL assays and Ki-67 staining (scale bar: 100 µm) (**i**)

The anti-tumor effect of BCP NPs in vivo were further evaluated since in view of perfect anti-tumor effect in vitro and non-toxic or side effects to organs in vivo. First step, the accumulation ability of PLGA nanoparticles in tumor site were verified by Caliper IVIS Lumina II. CY7@PLGA NPs were synthesized and injected intravenously to healthy or tumor bearing mice for the free BPA and free CPI-613 with no fluorescence. Fluorescence imaging was acquired at the corresponding time points. In different time points, the PLGA NPs had no obvious enrichment in the healthy group base on the fluorescence signal density. On the contrary, in the tumor bearing group, PLGA NPs were concentrated in tumor site quickly, the fluorescence signal reached to highest at 24 h then weaken slowly. At 48 h after injection. There were still a large number of particles accumulation in the tumor site, and a few residue in the lung, liver and kidney. The experiment demonstrated that PLGA nanoparticles could successfully accumulate in the tumor site through enhanced permeability and retention (EPR) effect to achieve the function of drug delivery. The carrier provides a basic guarantee for our anti-tumor experiment in vivo (Fig. 6a–c).

Next step, the anti-tumor activity of BCP NPs were evaluated in Hepa1–6 subcutaneous tumor bearing mice followed with the treatment scheme (Fig. 6d). As expected, the blank PLGA NPs group were similar to normal saline group, both unable to inhibit tumor growth. For free drugs group, the tumor volume was only increased by 1.05 times. For BPA NPs and CPI NPs groups, the tumor volume were 0.98 and 1.06 times of the original, respectively, which significantly inhibited the growth of tumor. Surprisingly, BCP NPs treated group not only completely inhibited tumor growth, but also significantly reduced tumor volume to 0.54 times of the original volume (Fig. 6f). In addition, the results can also be seen intuitively from the tumor image and tumor weight results (Fig. 6g). All the results showed that BCP NPs had a good synergistic effect in inhibiting tumor growth. Meanwhile, the body weight change of mice during the treatment can be ignored, testified again that the systemic toxicity of the drug is low (Fig. 6d and Additional file 1: Fig. S8).

At last, in order to fully understand the therapeutic effect of BCP NPs, paraffin section method was applied to conduct a detailed study on tumor tissue proliferation, apoptosis and lesion level after different treatment (Fig. 6i). H&E and Ki-67 results showed that compared with free drugs or single drug nanoparticles (BPA NPs and CPI NPs), the tumor growth and proliferation in BCP NPs group were significantly inhibited. TUNEL results showed the apoptotic cells was the largest in BCP group than other groups, which indicated that BCP NPs induced apoptosis of tumor cells and played an anti-tumor role (Fig. 6h, i). These data show that BCP NPs that co-loaded with 3-BPA and CPI-613 can induce apoptosis to die by inhibiting cell proliferation.

### In vivo combined treatment of BCP and STING agonists

**Fig. 7:**
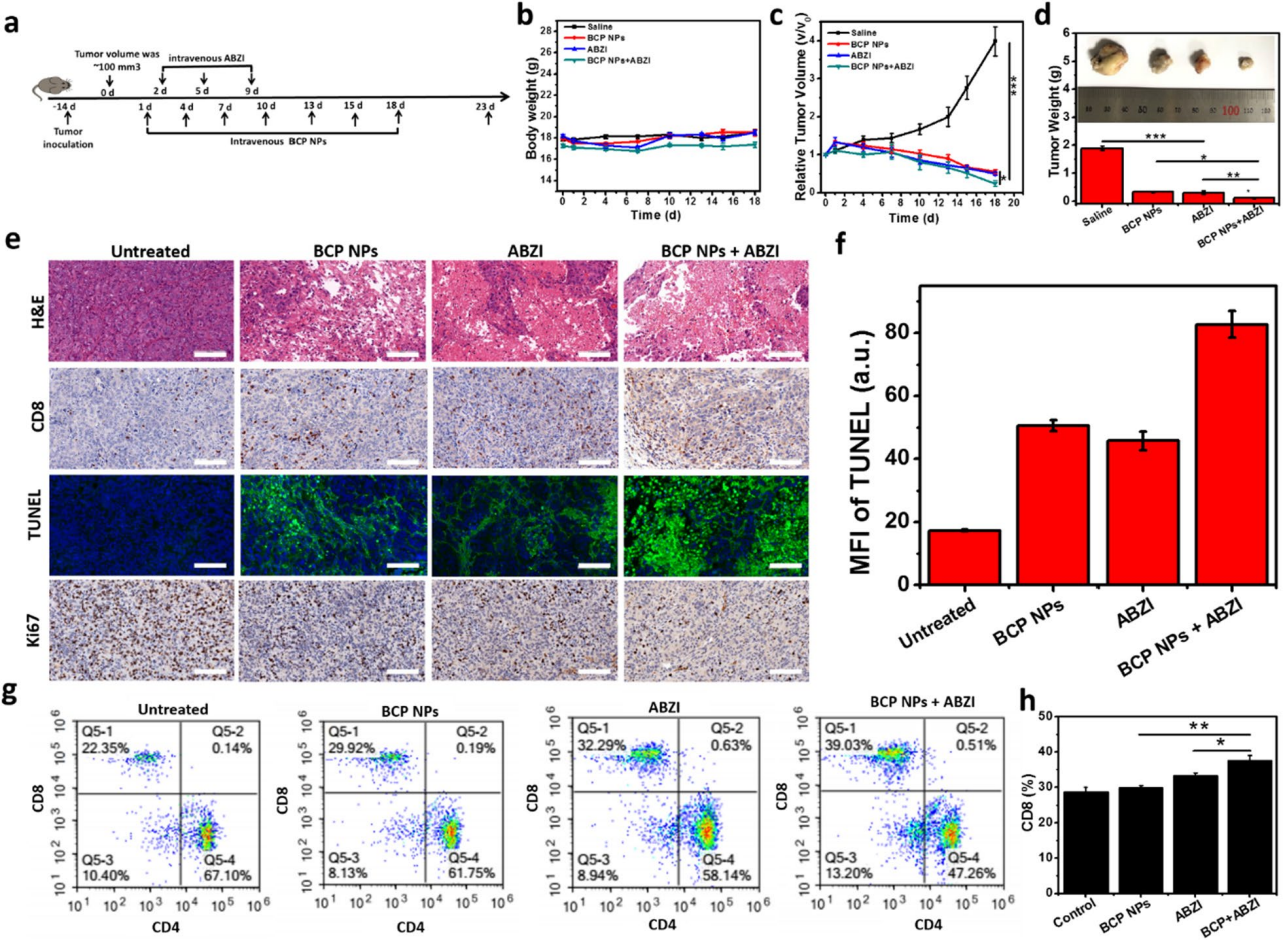
The combination of metabolic therapy and Sting agonists can enhance the anti-tumor effect. Schematic of treatment on a Hepa1–6 tumor model (**a**); Mouse body weight during the administration of different treatment protocols (**b**); Tumor volume variation during the administration of different treatment protocols (**c**); Weight of different resected tumors on day 18 (**d**); Tumor tissue section analysis of tumor sections by H&E staining, CD8^+^ T cells, TUNEL assays and Ki67 staining (**e**) (scale bar: 100 µm). The flow cytometry analysis of CD8^+^ T cells (**f**–**h**)

The signaling molecule: STING, which plays an important role in controlling the transcription of many host defense genes [28, 49, 50]. In recent years, due to the activation of cGAS-STING axis can accelerate cancer cell death, and the importance of cGAS-STING axis in regulating cancer immune cycle, many kinds of STING agonists have been developed to activate cGAS-STING pathway to achieve anti-cancer effect [51]. GSK has developed the first new non nucleotide sting agonist ABZI [30]. The agonist not only has strong antitumor effect, but also overcomes the disadvantages of intratumoral injection. As we found that BCP NPs can enhance the activation of STING, we further verified its anti-tumor effect in vivo when combination with sting agonists. As shown in Fig. 7a. The changes of body weight and tumor volume of mice in different groups during the treatment was monitored. The results showed that the weight of mice did not change much during the treatment, indicating that ABZI or BCP NPs had no obvious toxicity to mice. At the same time, the “Results” section showed that ABZI and BCP NPs had no obvious toxicity to major organs (Additional file 1: Fig. S9). The tumor volume of ABZI and BCP NPs treatment groups were 0.50 and 0.55 times of the original, respectively, yet the tumor volume of ABZI + BCP NPs combination group was 0.24 times of the original, and the tumor reduction degree was significant higher than that of ABZI small molecule group and BCP NPs alone group. It also could conclude that ABZI + BCP group has better anti-tumor effect than others (Fig. 7c, d). These results suggest that ABZI and BCP NPs have the effect of combined treatment. At the same time, H&E results showed that the nuclei of tumor cells in ABZI + BCP NPs treatment group were significantly shrunk, and the number of cells became less dense. TUNEL staining also showed that the green fluorescence was the strongest in the ABZI + BCP NPs treatment group compared with other groups, indicating that the proportion of apoptotic cells was more. Ki-67 results also showed that ABZI + BCP NPs treatment group had the most significant inhibition of tumor cell proliferation (Fig. 7e, f). The CD8^+^ T cell numbers after administration were calculated by flow cytometry. Compared with untreated group, the CD8^+^ T cells in all other groups increased to some extent. What’s more, the BCP NPs + ABZI treated group had the most CD8^+^ T cell number. And there was significant difference between the ABZI and the BCP NPs + ABZI treated group, which demonstrated that the BCP NPs can enhance the immune activation ability of ABZI agonist, so as to enhance the anti-tumor effect (Fig. 7g–f).

## Discussion

Eliminating tumors by blocking the energy metabolism of it is a new strategy different from traditional therapy approach. The synergistic effects of 3-BPA and CPI-613 have not been studied due to the dissociability and insolubility of the two drugs, respectively. To solve this problem, we first proposed to study the synergistic effect of the two drugs and chose PLGA as nanocarrier to load these two drugs at the same time. We first screened out the most significant synergistic drug ratio (1:1). Then the optimized proportion of BCP NPs was selected to explore its anti-hepatoma effect.

Our study suggested that BCP NPs not only have a significant synergistic anti-hepatoma effect but also have no obvious toxic and side effects in vivo and vitro, which has good biological safety. Compared with other treated groups, BCP NPs showed strong synergistic effect. It was found that BCP NPs decreased the contents of ATP and lactate and the consumption of glucose in Hepa1–6 cells. Meanwhile, metabolism-related genes and proteins in tumor cell were significantly decreased. The synergistic anti-tumor effect of BCP NPs was also confirmed in the subcutaneous tumor model of Hepa1–6 cells.

Moreover, we first identify BPC NPs could activate STING signal pathway and reveal the combination of BCP NPs and STING agonist inhibited the growth of tumor cells to a greater extent, the tumor volume even decreased to 0.24 times of the original after treatment.

## Conclusion

In this study, we successfully improved the dosage forms of two small molecule metabolic inhibitors by using nano carriers, and found that the synthesized BCP nanoparticles have synergistic antitumor effect. In addition, we found that the nanoparticles can also activate innate immunity, and significantly increase the antitumor ability in vivo after combined with sting agonists. This groundbreaking research will help overcome the limitations of the combination of multiple antitumor drugs and inspire the future research of metabolic therapy, immunotherapy and their combination in the treatment of tumors.

## Materials and methods

### Materials

3-BPA (bromopyruvic acid) and CPI-613(6,8-bis-benzylsulfanyl-octanoic acid) were purchased from Acros (UK). PLGA (Mw = 38–54 kg/mol) was acquired from Jinan Dai Gang Biomaterial Co., Ltd (China). Poly(vinyl alcohol) 1788 (PVA, low molecular weight), d-(+)-trehalose anhydrous and dichloromethane were obtained from Aladdin Reagent (China). DMEM was purchased from biosharp (China). Fetal bovine serum (FBS) was acquired from Cellma (China). Dimethyl sulfoxide (DMSO) were obtained from BioFroxx (China). 3-(4,5-Dimethylthiazol-2-yl)-thiazolyl blue tetrazolium bromide (MTT) was provided by GoldBio. Coumarin 6 was acquired from Sigma-Aldrich (USA). Alexa Fluor^®^ 488 annexin V/Dead Cell Apoptosis Kit was purchased from Thermo Fisher Scientific (USA). Mitochondrial Membrane Potential Assay Kit with JC-1 was obtained from Solarbio life sciences Co., Ltd (China). Pyruvate assay kit and glucose kit were obtained from Nanjing jiancheng Co., Ltd (China). ATP Assay Kit was obtained from Beyotime. Lactic acid kit, β-actin, GAPDH and TBK1 antibody were obtainted from Abclonal (China). HK-II antibody was purchased from proteintech. c-Myc antibody was purchased from Beyotime (China). P-TBKI antibody were provided by Cell Signaling Technology. STING antibody was donated by HongbinShu’s laboratory.

### Preparation of CPI-613 and 3-BPA loaded PLGA NPs

The PLGA nanoparticles were obtained by the classic emulsion solvent evaporation approach. Briefly, PLGA (100 mg) and different amounts of CPI-613 were added to dichloromethane (DCM)-methanol (2 mL) which was considered as the oil phase. Meanwhile, 3-bromopyruvate was dissolved in 150 µL deionized water, which was considered as the aqueous phase. Then, the aqueous phase solution was added to the dichloromethane oil phase drop by drop under the eddy condition. Subsequently, the emulsion was added drop by drop intoPVA aqueous solution (4 mL, 5%). Then placed it into the ice bath for ultrasound with a Sonifier 450 (Branson Sonic Power, Danbury, CT, USA) immediately. After the ultrasound, the emulsion was quickly poured into PVA aqueous solution (50 mL 0.05%). The organic solvent dichloromethane in emulsion was evaporated by accelerated agitation at room temperature. Then, the (BPA +CPI)@PLGA NPs (BCP NPs) were dried by a lyophilizer, and stored at − 20 °C after the centrifugation at 12,000*g*.

### MTT assay and synergy analysis

MTT assay were applied to explore the biocompatibility and cytotoxicity of PLGA NPs and BCP NPs in vitro. Briefly, Hepa1–6 cells (1 × 10^5^ mL^−1^) were seeded on a 96-well culture plate (100 μL per well) and incubated in Cell incubator at 37 °C with 5% CO_2_. After incubating for 24 h, add the different drugs with different concentration, DMEM without drugs and 1% Triton were used as negative control and positive control respectively. Then, incubation for another 24 h or 48 h respectively, washed with PBS and added MTT solution (100 µL per well, 0.5 mg/mL). After further incubation of 4 h, DMSO (200 µL per well) was added to dissolve formazan crystals, then shaked for 15 min gently. The optical absorbance at 570 nm was measured by a microplate reader (spectramax i3, Molecular Devicesmd).

To explore the combined therapeutic index of nanoparticles with different drug ratios, we used CalcuSyn software 1.0 (Biosoft, Cambridge, UK) o calculate the CI value and the median-effect equation. Then plotted the CI value versus Fa to determine the occurrence ratio dependent synergy. (CI > 1, antagonism; CI = 1, additivity; and CI < 1, synergy).

### Characterizations

To determine the morphology of the BCP NPs, a transmission electron microscope (TEM, JEM-2100, JEOL, Japan) was used. Meanwhile, a dynamic light scattering (DLS, Malvern ZS90) was used to measure the hydrodynamic size and zeta potential. To determine the crystalline forms of the BCP NPs, an X-ray diffractometer (XRD-7000, Shimadzu, Japan) was used. The quantification of CPI-613 and 3-BPA were carried out by the high performance liquid phase (HPLC, Agilent, USA) and pyruvate assay kit.

### HPLC analysis

The content of CPI-613 in PLGA was analyzed by HPLC. The mobile phase was methanol water (85:15 V/V), the detection slope length was 215 nm, and the flow rate was 1 mL/min. The range of the standard curve was 4780–0.478 μg/mL. In order to remove the non encapsulated free drug and fully release the drug encapsulated in PLGA nanoparticles, so as to obtain the accurate entrapment efficiency and drug loading capacity of drug loaded PLGA nanoparticles, we put the nanoparticle suspension just volatilized dichloromethane in a centrifuge tube, then centrifuged at 12,000 rpm for 20 min, discarded the supernatant, washed with water for three times. After freeze-drying, 5 mg of BCP NPs was dissolved with methanol, absorb the supernatant and detect them by HPLC.

### Drug release and intracellular NPs uptake

We used the model drug Coumarin 6 and hydrophilic Cy7 to replaced CPI-613 and 3-BPA to determine drug relase and intracellular NPs uptake. Briefly, the PLGA NPS loaded with Coumarin 6 and Cy7 were dissolved with 5 mL PBS (1 mg/mL). The suspension was transferred into a 15 mL centrifuge tube and put the centrifuge tube in an air bath shaking at 220 rpm at 37 °C. 1 mL PBS was taken out to centrifuge at 12,000 rpm for 15 min at specific time points. Then saved the supernatant for measurement and added 1 mL fresh release medium to resuspended the precipitation, add it to the original 15 mL centrifuge tub. The amount of Coumarin 6 and Cy7 were measured by fluoremetry with a microplate reader (spectramax i3, Molecular Devices).

### In vitro cell apoptosis

To explore the toxicity of the BCP NPs, Hepa1–6 cells (1.5 × 10^5^ cells per well) were seeded onto a 12-well plate and cultured for 24 h. free CPI-613 (50 µM), free 3-BPA (50 µM), CPI-613 NPs (50 µM), 3-BPA NPs (50 µM) and BCP NPs (total drugs: 50 µM) were added into each well. After 24 h or 48 h, cells were washed with PBS, and stained with a LIVE/DEAD Viability/Cytotoxicity Kit and observed under a fluorescence microscope (NIKON ECLIPSE Ts2).

The Alexa Fluor 488 annexin V-FITC and JC-1 staining was Carried out accordied to the instructions. Then observed confocal microscope (LSM880, ZEISS) or flowmetry analysis.

### Western blots

Hepa1–6 cells and AML12 cells (1.3 × 10^5^ cells per well) were seeded onto a 24-well plate and cultured for 12 h. 3-BPA NPs (50 µM), CPI-613 NPs (50 µM), and BCP NPs (total drugs: 50 µM) were added into each well. Then incubated for 24 h. Cells were washed with PBS and lysed in TAP lysis buffer for 30 min at 4 °C. Protein samples were resolved by SDS-PAGE. transferred to Nitrocellulose membranes (Bio-Rad, 1620177). Membrane was blocked in 5% no fat milk for 1 h, and incubated with primary antibody at 4 °C overnight. After washed with PBS-T. The membranes were then incubated with secondary antibody at room temperature for 1 h. Then developed in darkroom with ECL substrate.

### Real-time PCR

Totoal RNA extraction was according to the RNAiso Plus kit instruction (TAKARA, 9108). 1 µg total RNA was transcribed into cDNA using MonScript™ RTIII Super Mix with dsDNase (Monad Biotech Co., Ltd. MR05201). Fluorescence quantitative PCR reaction was performed by Real time fluorescence quantitative PCR instrument (Bio-Rad, CFX96). The comparative Ct method was used to determine relative mRNA expression of genes as normalized by the housekeeping genes β-actin. Primer sequences as following. Murine β-actin, 5ʹ-GGCTGTATTCCCCTCCATCG-3ʹ (forward) and 5ʹ-CCAGTTGGTAACAATGCCATGT-3ʹ (reverse); Murine GAPDH, 5ʹ-AGGTCGGTGTGAACGGATTTG-3ʹ (forward) and 5ʹ-TGTAGACCATGTAGTTGAGGTCA-3ʹ (reverse); Murine HK2, 5ʹ-TGATCGCCTGCTTATTCACGG-3ʹ (forward) and 5ʹ-AACCGCCTAGAAATCTCCAGA-3ʹ (reverse); Murine c-Myc, 5ʹ-ATGCCCCTCAACGTGAACTTC-3ʹ (forward) and 5ʹ-CGCAACATAGGATGGAGAGCA-3ʹ (reverse); Murine MCT1, 5ʹ-TGTTAGTCGGAGCCTTCATTTC-3ʹ (forward) and 5ʹ-CACTGGTCGTTGCACTGAATA-3ʹ (reverse); Murine LDHA, 5ʹ-TGTCTCCAGCAAAGACTACTGT-3ʹ (forward) and 5ʹ-GACTGTACTTGACAATGTTGGGA-3ʹ (reverse); Murine IFN-β, 5ʹ-CAGCTCCAAGAAAGGACGAAC-3ʹ (forward) and 5ʹ-GGCAGTGTAACTCTTCTGCAT-3ʹ (reverse).

### Mice samples

All mice were purchased from Wuhan Institute of Biological Products Co. Ltd and Beijing Vital River Laboratory Animal Technology Co., Ltd. 6–8 weeks female C57BL/6 mice were used for experiments. All studies were approved by the Institutional Animal Care and Use Committee (IACUC) of Medical Research Institute, Wuhan University.

### Routine hematology study in vivo

To evaluate the biocompatibility and potential immunogenicity, Blood routine and liver and kidney function were analyzed. The whole blood samples were collected from BALB/c mice, detected by automatic blood cell analyzer (BC-2800vet, Mindray, China) and automatic biochemical analyser (Chemray 240, Shenzhen Redu Life Technology, China).

### In vivo biocompatibility assay

The biocompatibility of BCP NPs was explored by hemolysis test. Briefly, to obtain the pure erythrocytes, the whole blood samples that obtained from healthy C57BL/6 mice were washed five times with PBS by centrifuged at 3000 rpm for 5 min. The red blood cells were re-suspended in PBS for later use. BCP NPs dispersed in PBS at various concentrations (0.5, 1, 2, 5, 10, 20, 50 and 100 µM), and incubated at 37 °C. 100% hemolysis was deemed as the erythrocytes mixed with DI water. The erythrocytes mixed with PBS was regarded as positive control. Finally, optical absorbance at 540 nm was recorded by a microplate reader.

### Establishment of 4T1 tumor model

To establish the Hepa1–6 hepatocellular carcinoma tumor model, C57BL/6 mice were subcutaneously with Hepa1–6 cells resuspended in DMEM (4 × 10^6^ cells per mouse) on the right abdomen.

### In vivo fluorescence imaging

CY7 was used to track the nanoparticles in tumor-bearing mice. Briefly, tumor-bearing C57BL/6 mice were given the PLGA NPs which loaded Cy7 intravenously at a concentration of 5 mg/kg. Normal mice were used as negative control. The signal of Cy7 was measured at appropriate time points with the Caliper IVIS Lumina II (IVIS, Xenogen, Caliper Instruments).

### Tumor inhibition in vivo

Tumor volume was measured in real time and calculated based on Eq. (1) [48] as follows:

1 $${\text{Tumor volume}} = \left( {\text{tumor length}} \right) \times \left( {\text{tumor width}} \right)^{{2}} \times 0.{5},$$

When the tumor size reached approximately 100 mm^3^. Mice were randomly divided into six groups, and were intravenously injected with 100 µL of (1) saline, (2) PLGA NPs, (3) free drugs (3-BPA + CPI-613), (4) BPA NPs, (5) CPI NPs, and (6) BCP NPs. The equivalent total drug dosages of 15 mg/kg were used for all the groups, respectively. An injection of medicine was given every 3 days. Simultaneously. The tumor volume was and body weight were recorded every other day. Finally, sacrificed the mice and excised the tumors and organs. The hematoxylin and eosin (H&E) staining and immunofluorescent staining were all according to the standard protocols.

### The combination of NPs and STING agonist

When average tumor volume was 100 mm^3^, mice were randomly divided into four groups, 10 mice each group. Mice were intravenously injected with 100 µL of (1) saline, (2) BCP NPs, (3) ABZI (STING agonist), and (4) BCP NPs + ABZI. For all the groups, the total drug dosages were equivalent. The total drugs of BCP NPs were 15 mg/kg, and was given every 3 days. Meanwhile, the dose of ABZI was 1.5 mg/kg. The mice’s body weight and tumor volume were measured daily. The hematoxylin and eosin (H&E) staining and immunofluorescent staining were all according to the standard protocols [52].

To explore whether ABZI activated the immune system in mice, the changes of CD8 molecules on the surface of mouse T cells after administration was examined. Briefly, all spleen samples were processed to cell suspension, re-suspended in the flow buffer, and incubated with mouse Fc blocker. Finally, before the flow cytometry analysis, cells were stained with CD45-PE, CD3-V421, and CD8-APC.

### Statistical analysis

All statistical analyses were completed by one-way analysis of variance (ANOVA). A P-value smaller than 0.05 (*P < 0.05, **P < 0.01, ***P < 0.001) was regarded as statistically significant. All the data analyses were used Origin (OriginLab, MA, USA) and shown as the mean ± standard deviation (SD).

## Supplementary Information

**Additional file 1: Table S1. **Z-average and zeta potential of different CPI-613/3-BPA PLGA NPs. **Table S2. **Characteristics of the CPI-613/3-BPA-loaded nanoparticles. **Table S3. **Table 1 IC_50_ (μM, total drugs concentration) of nanoparticles against Hepa1–6 cells line. **Figure S1.** AML 12 cells treated with BCP NPs under gradient concentrations for 24 h and 48 h. **Figure S2. **4T1 cells (a) and MCF-7 cells (b) treated with BCP NPs under gradient concentrations for 24 h and 48 h. **Figure S3. **Stability of nanoparticles under different conditions. **Figure S4. **Fluorescent images of Hepa1–6 cells after being treated with BCP NPs (equivalent Coumarin concentration: 10 µg/mL) for 1, 2, 4 and 6 h (scale bar: 100 µm). **Figure S5. **Fluorescence images of Hepa1–6 cells after various treatments analyzed by a LIVE/DEAD viability assay. The green and red dots denote live and dead cells, respectively (scale bar: 500 µm). **Figure S6. **Western blot analysis of phosphorylated TBK1 after the treatment of ABZI and BCP NPs of Hepa1–6 cells (a); The heat map for mRNA expression of metabolism-related genes of GAPDH, c-Myc, MCT1, HK-II, PKM, LDHA when the Hepa1–6 cells treated with different drugs (b). **Figure S7. **Hemolysis rate by incubating RBCs with DI water (positive control), PBS (negative control) or BCP NPs under various concentrations. (Inset: corresponding digital photos of centrifuge tube containing different samples). **Figure S8. **H&E stained tumor slices excised from major organs after the mice receiving various treatments (scale bar: 100 µm). **Figure S9. **H&E stained tumor slices excised from major organs after the mice receiving various treatments (scale bar: 100 µm).

## Data Availability

The datasets generated and/or analyzed during the current study are available from the corresponding authors on reasonable request.
