# Supplementary material for: Cocktail strategy based on a dual function nanoparticle and immune activator for effective tumor suppressive
Source: J Nanobiotechnology. 2022 Feb 17;20:84. doi: 10.1186/s12951-022-01241-y (PMC8851817; doi:10.1186/s12951-022-01241-y)
Supplement: Supplementary file 1 — Additional file 1: Table S1. Z-average and zeta potential of different CPI-613/3-BPA PLGA NPs. Table S2. Characteristics of the CPI-613/3-BPA-loaded nanoparticles. Table S3. Table 1 IC50 (μM, total drugs concentration) of nanoparticles against Hepa1–6 cells line. Figure S1. AML 12 cells treated with BCP NPs under gradient concentrations for 24 h and 48 h. Figure S2. 4T1 cells (a) and MCF-7 cells (b) treated with BCP NPs under gradient concentrations for 24 h and 48 h. Figure S3. Stability of nanoparticles under different conditions. Figure S4. Fluorescent images of Hepa1–6 cells after being treated with BCP NPs (equivalent Coumarin concentration: 10 µg/mL) for 1, 2, 4 and 6 h (scale bar: 100 µm). Figure S5. Fluorescence images of Hepa1–6 cells after various treatments analyzed by a LIVE/DEAD viability assay. The green and red dots denote live and dead cells, respectively (scale bar: 500 µm). Figure S6. Western blot analysis of phosphorylated TBK1 after the treatment of ABZI and BCP NPs of Hepa1–6 cells (a); The heat map for mRNA expression of metabolism-related genes of GAPDH, c-Myc, MCT1, HK-II, PKM, LDHA when the Hepa1–6 cells treated with different drugs (b). Figure S7. Hemolysis rate by incubating RBCs with DI water (positive control), PBS (negative control) or BCP NPs under various concentrations. (Inset: corresponding digital photos of centrifuge tube containing different samples). Figure S8. H&E stained tumor slices excised from major organs after the mice receiving various treatments (scale bar: 100 µm). Figure S9. H&E stained tumor slices excised from major organs after the mice receiving various treatments (scale bar: 100 µm). [file 12951_2022_1241_MOESM1_ESM.docx]

**Additional information**

**Cocktail strategy based on a dual function nanoparticle and immune activator for effective tumor suppressive**

Qian Li^1,2,3，#^, Qiubing Chen^1,2,3,#^, Xue Yang^1,2^, Yuelan Zhang^1,2^, Linyue Lv^1,2^, Zhuyou Zhang^1,2^, Shaowei Zeng^1,2^, Jiaxi Lv^4^, Sijin Liu^5^*, Bishi Fu^1,2,3^*

^1^ Department of Paediatrics, State Key Laboratory of Virology, Frontier Science Center for Immunology and Metabolism, Medical Research Institute, Zhongnan Hospital of Wuhan University, Wuhan, China

^2^ Department of Pulmonary and Critical Care Medicine, Zhongnan Hospital of Wuhan University

^3^ Wuhan Research Center for Infectious Diseases and Cancer, Chinese Academy of Medical Sciences, Wuhan, China

^4^ Department of Clinical Medicine，Fourth Clinical Medical College, Capital Medical University, Beijing, P.R. China

^5^ State Key Laboratory of Environmental Chemistry and Ecotoxicology, Research Center for Eco-Environmental Sciences, Chinese Academy of Sciences, Beijing, China

^#^ Q. Li and Q. Chen contributed equally to this paper.

* Corresponding author to B. Fu: bishi_fu@whu.edu.cn

**Table S1** Z-Average and Zeta Potential of different CPI-613/3-BPA PLGA NPs

**Table S2** Characteristics of the CPI-613/3-BPA-loaded nanoparticles

**Table S3** Table 1 IC_50_ (μM, total durgs concentration) of nanoparticles against Hepa1-6 cells line

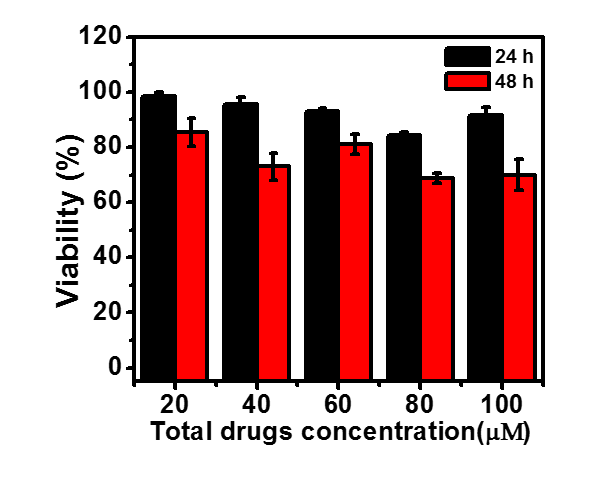


**Figure S1** AML 12 cells treated with BCP NPs under gradient concentrations for 24 h and 48 h.


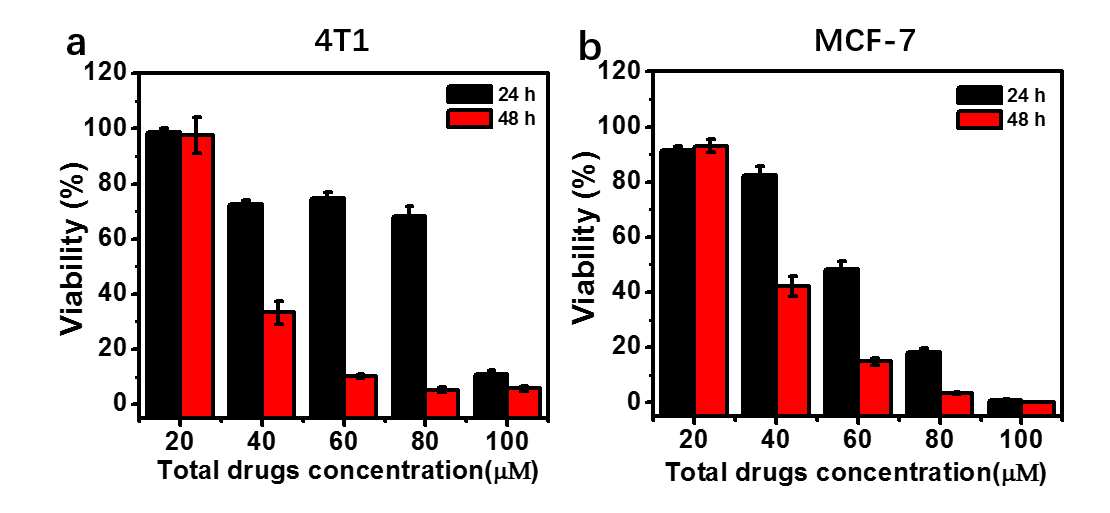


**Figure S2** 4T1 cells (a) and MCF-7 cells (b) treated with BCP NPs under gradient concentrations for 24 h and 48 h.


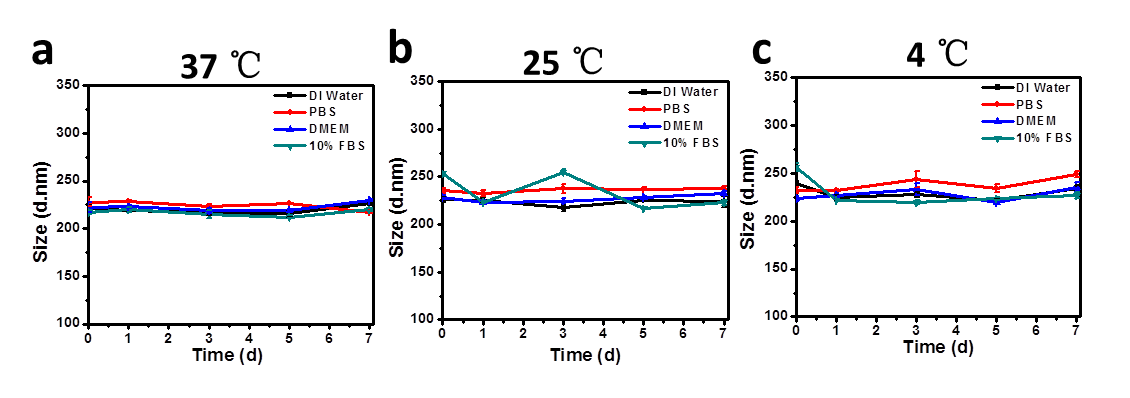


**Figure S3** Stability of nanoparticles under different conditions


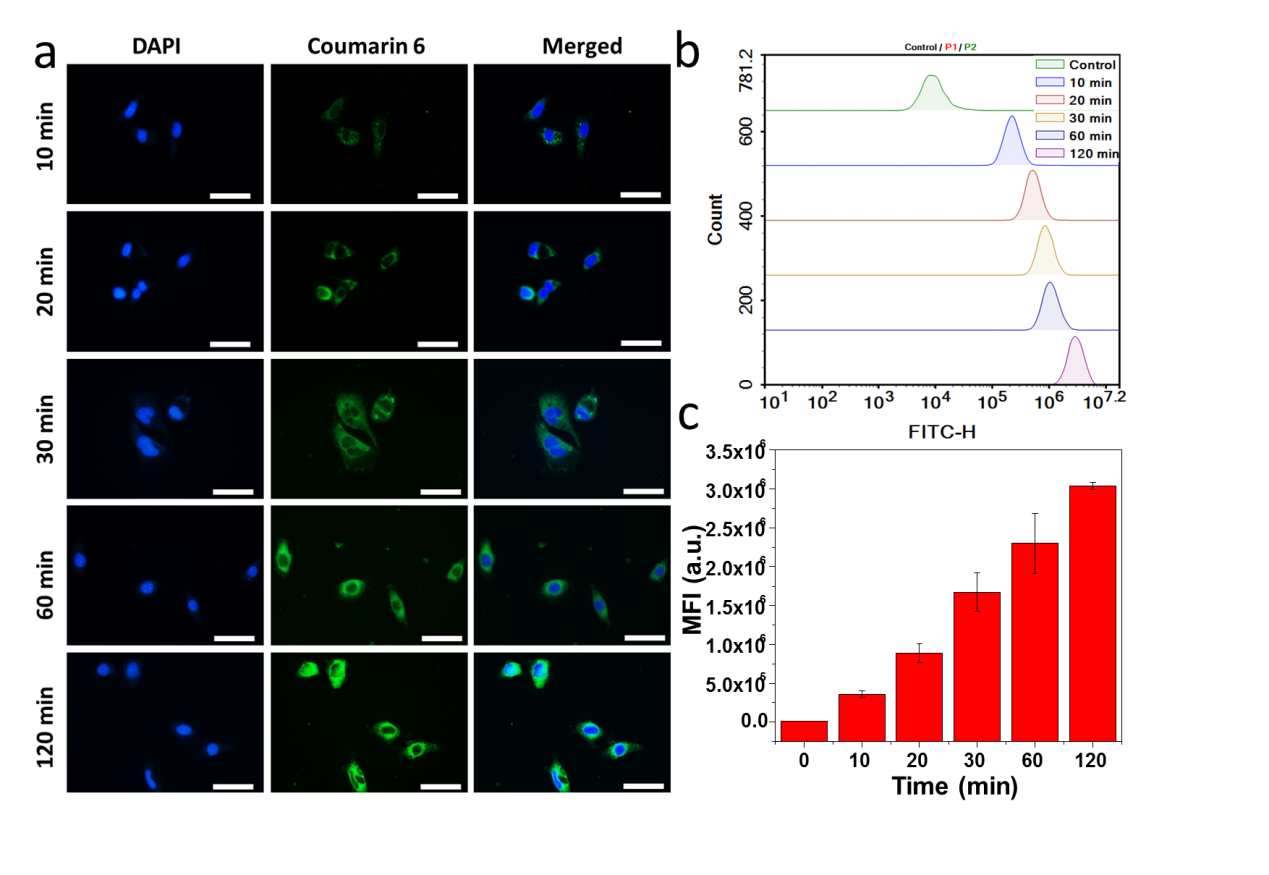


**Figure S4** Fluorescent images of Hepa1-6 cells after being treated with BCP NPs (equivalent Coumarin concentration: 10 µg·mL^-1^) for 1, 2, 4 and 6 h (scale bar: 100 µm).


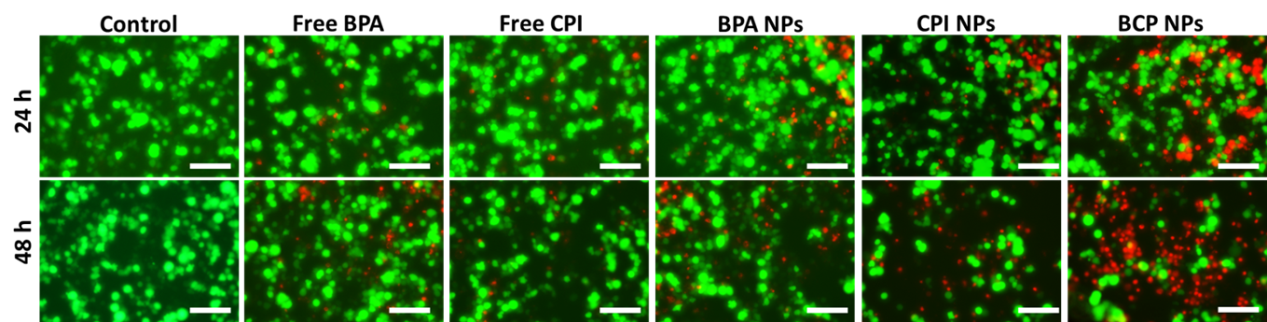


**Figure S5** Fluorescence images of Hepa1-6 cells after various treatments analyzed by a LIVE/DEAD viability assay. The green and red dots denote live and dead cells, respectively (scale bar: 500 µm).


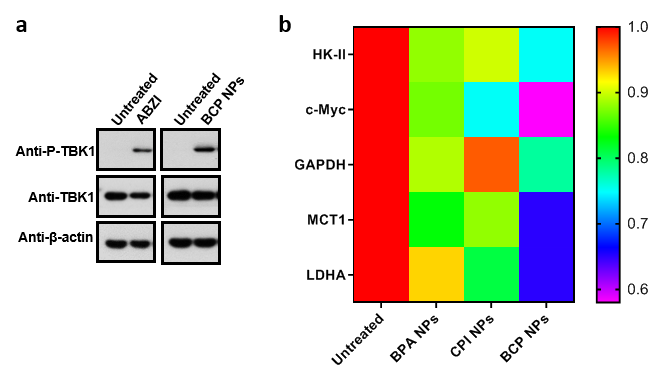


**Figure S6** Western blot analysis of Phosphorylated TBK1 after the treatment of ABZI and BCP NPs of Hepa1-6 cells (a); The heat map for mRNA expression of metabolism-related genes of GAPDH, c-Myc, MCT1, HK-II, PKM, LDHA when the Hepa1-6 cells treated with different drugs (b).


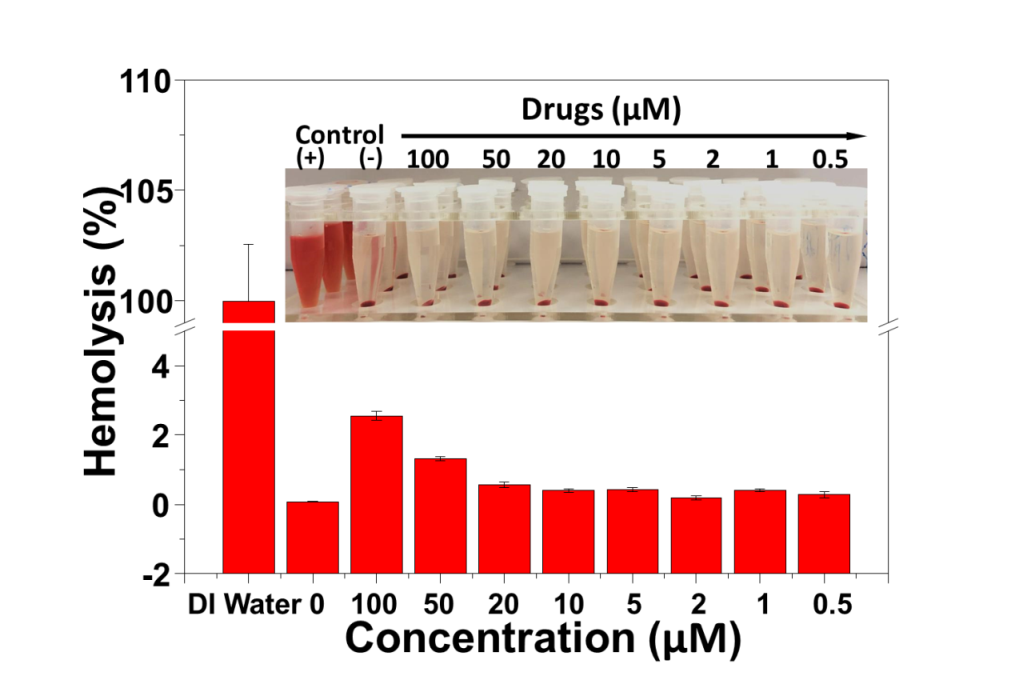


**Figure S7** Hemolysis rate by incubating RBCs with DI water (positive control), PBS (negative control) or BCP NPs under various concentrations. (Inset: corresponding digital photos of centrifuge tube containing different samples)


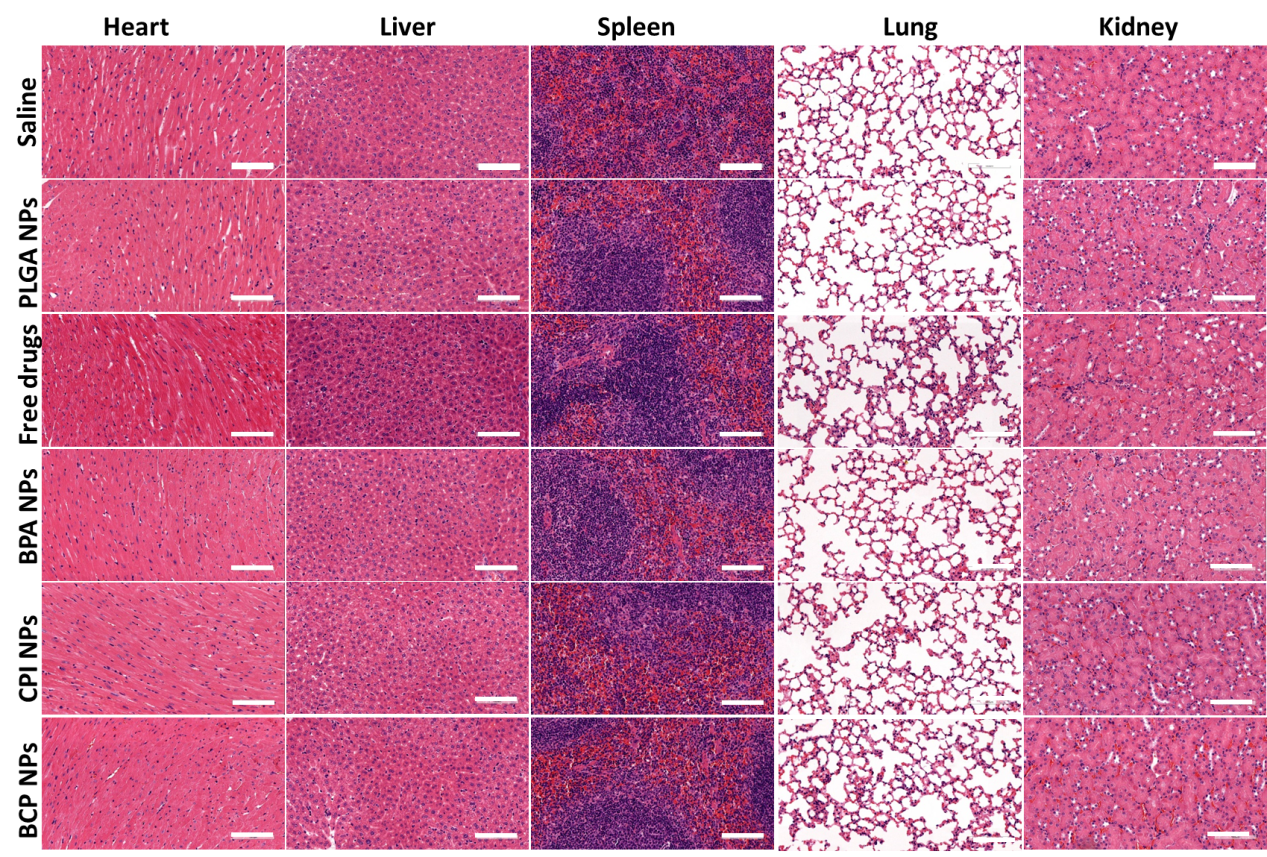


**Figure S8** H&E stained tumor slices excised from major organs after the mice receiving various treatments (scale bar: 100 µm).


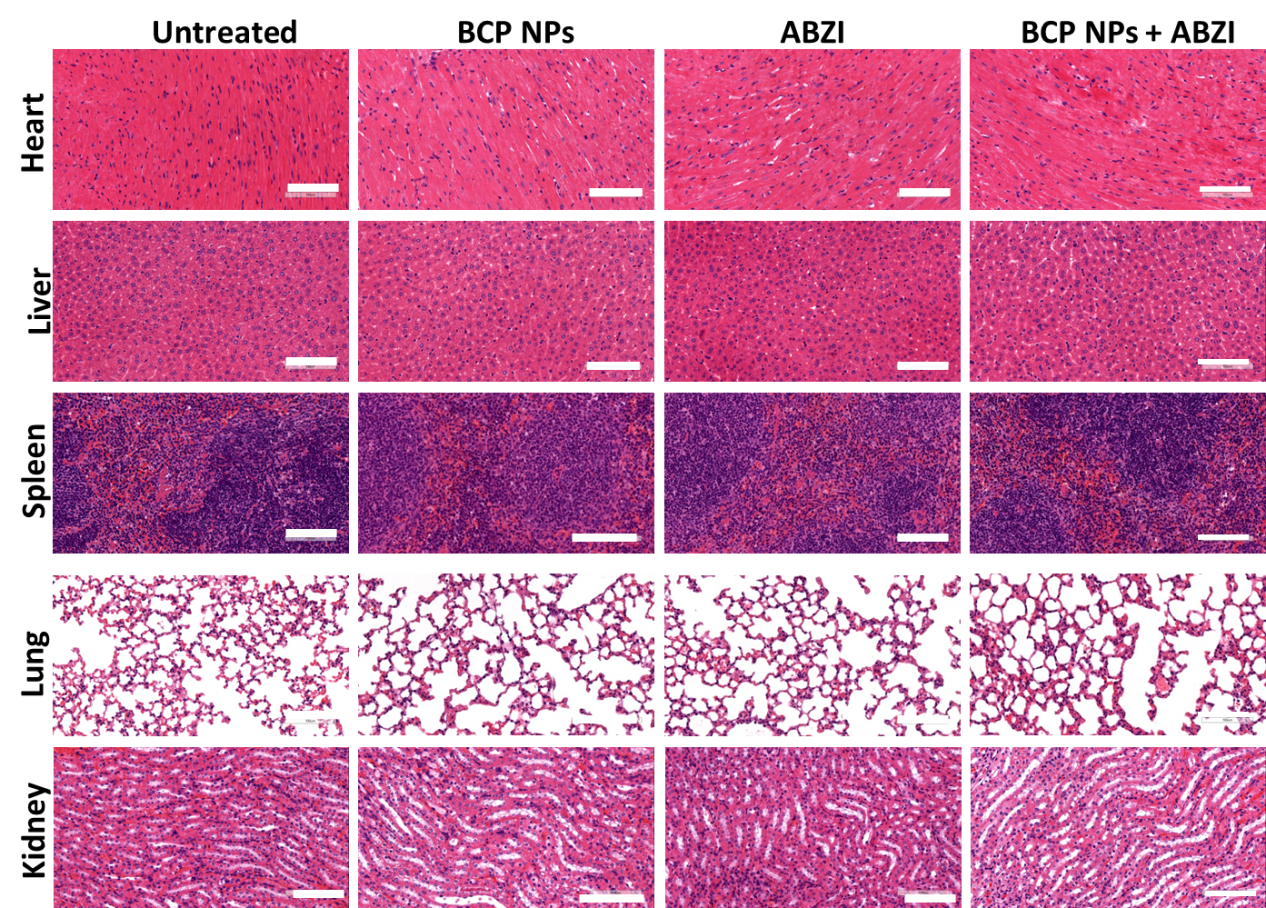


**Figure S9** H&E stained tumor slices excised from major organs after the mice receiving various treatments (scale bar: 100 µm).
